# Understanding the transfer and persistence of antimicrobial resistance in aquaculture using a model teleost gut system

**DOI:** 10.1186/s42523-025-00377-0

**Published:** 2025-02-22

**Authors:** Alexandru S. Barcan, Joseph L. Humble, Sandeep Kasaragod, Mohammad Saiful Islam Sajib, Rares A. Barcan, Philip McGinnity, Timothy J. Welch, Brendan Robertson, Emanuel Vamanu, Antonella Bacigalupo, Martin S. Llewellyn, Francisca Samsing

**Affiliations:** 1 SBOHVM, University of Glasgow, Graham Kerr Building, Glasgow, G12 8QQ UK; 2 Sydney School of Veterinary Science, The University of Sydney, Camden, NSW 2570 Australia; 3 School of Biological, Earth and Environmental Sciences, University College Cork, Cork, Ireland; 4 U.S. Department of Agriculture/Agricultural Research Service, National Center for Cool and Cold Water Aquaculture, Leetown, WV 25430 USA; 5 Maths & Physical Sciences, University of Sussex, Falmer, Brighton, BN1 9RH UK; 6 Faculty of Biotechnology, University of Agricultural Sciences and Veterinary Medicine, 011464 Bucharest, Romania; 7 MRC-University of Glasgow Centre for Virus Research, Glasgow, UK

## Abstract

**Background:**

The development, progression, and dissemination of antimicrobial resistance (AMR) are determined by interlinked human, animal, and environmental drivers, which pose severe risks to human and livestock health. Conjugative plasmid transfer drives the rapid dissemination of AMR among bacteria. In addition to the judicious use and implementation of stewardship programs, mitigating the spread of antibiotic resistance requires an understanding of the dynamics of AMR transfer among microbial communities, as well as the role of various microbial taxa as potential reservoirs that promote long-term AMR persistence. Here, we employed Hi-C, a high-throughput, culture-free technique, combined with qPCR, to monitor carriage and transfer of a multidrug-resistent (MDR) plasmid within an Atlantic salmon in vitro gut model during florfenicol treatment, a benzenesulfonyl antibiotic widely deployed in fin-fish aquaculture.

**Results:**

Microbial communities from the pyloric ceaca of three healthy adult farmed salmon were inoculated into three bioreactors simulating the teleost gut, which were developed for the SalmoSim gut system. The model system was then inoculated with the *Escherichia coli* strain ATCC 25922 carrying the plasmid pM07-1 and treated with florfenicol at a concentration of 150 mg/L in fish feed media for 5 days prior to the washout/recovery phase. Hi-C and metagenomic sequencing identified numerous transfer events, including those involving gram-negative and gram-positive taxa, and, crucially, the transfer and persistence of the plasmid continued once florfenicol treatment was withdrawn.

**Conclusions:**

Our findings highlight the role of the commensal teleost gut flora as a reservoir for AMR even once antimicrobial selective pressure has been withdrawn. Our system also provides a model to study how different treatment regimens and interventions may be deployed to mitigate AMR persistence.

**Supplementary Information:**

The online version contains supplementary material available at 10.1186/s42523-025-00377-0.

## Background

Antibiotics have existed for hundreds of millions of years, and the phenomenon of resistance has developed alongside them [1]. Throughout history, microorganisms have evolved various mechanisms to evade the challenges posed by chemical agents and environmental pressures, including those from antimicrobial drugs. The acquisition of resistance traits is a natural and adaptive process for microbes, enabling them to survive in the presence of antimicrobial agents [2]. In any bacterial population, a small number of individuals naturally exhibit resistance to specific antimicrobial compounds. When these substances are introduced, they eliminate most susceptible bacteria, sparing those with resistance genes [3]. These individuals then reproduce, leading to a population predominantly composed of resistant bacteria over time. This process represents a straightforward example of the survival of natural selection. However, complexity increases with the ability of bacteria to exchange genetic material, not only among their kind but also across different taxa [4].

Bacterial DNA, including genes responsible for antimicrobial resistance (AMR), can be transferred through various mechanisms, such as conjugation, transposition, and transformation, which are the most well documented [5]. The transmission of antibiotic resistance genes both within and between species predominantly occurs through plasmids. Plasmids are mobile, typically circular genetic elements ranging in size from thousands to hundreds of thousands of base pairs, and can replicate independently of the host’s chromosome [6].

Through these exchanges of genetic material, resistance factors can migrate from harmless bacteria to those that pose significant threats to animals or humans, complicating or, in some instances, rendering the treatment of these diseases impossible [7]. In addition to the transfer of AMR genes, other genetic elements related to virulence and stress resistance can also be disseminated through these mechanisms, potentially transforming nonpathogenic bacteria into harmful pathogens [8]. This phenomenon is especially relevant in the microbiome of vertebrate guts, which accelerates the emergence of antibiotic-resistant bacterial strains and facilitates the proliferation of virulence factors [9]. Compared with other bacterial environments, the gut microbial ecosystem is crowded and dense, making it easier for bacteria to swap genes across species via horizontal transfer. This high level of gene exchange leads to important genetic diversity for microbes [10].

The presence of antimicrobial resistance genes in aquaculture is a complex, two-way process that poses significant risks to both the environment and human health. Wastewater from various sources, including residential, industrial, and agricultural runoff, often contains AMR genes [11]. When this contaminated wastewater enters aquaculture systems, which are generally located downstream from urban environments, these AMR genes can be introduced into the fish farming environment. Within this aquaculture environment, AMR genes can persist and proliferate, leading to the development of resistant bacterial populations, which can then spread and potentially infect other fish, contaminating the entire system. The issue does not stop there; it extends beyond the boundaries of the fish farm. Effluents and runoff from these aquaculture farms can carry AMR genes back into surrounding natural water bodies, creating a feedback loop in which resistance spreads further into the environment. This domino effect can impact wild aquatic life, leading to biodiversity loss and the potential for resistant bacteria to enter the food web. Moreover, when humans consume seafood contaminated with AMR genes, they risk contracting resistant infections that can result in illness or death. Recreational and occupational exposure to contaminated water also poses health risks, as people can come into contact with resistant bacteria. Aquaculture, one of the world's fastest-growing food production industries, remains the least researched field in AMR [12]. Recent research has highlighted the prevalence of antibiotic resistance in aquaculture-associated pathogens, such as Vibrio species [13].

**Fig. 1:**
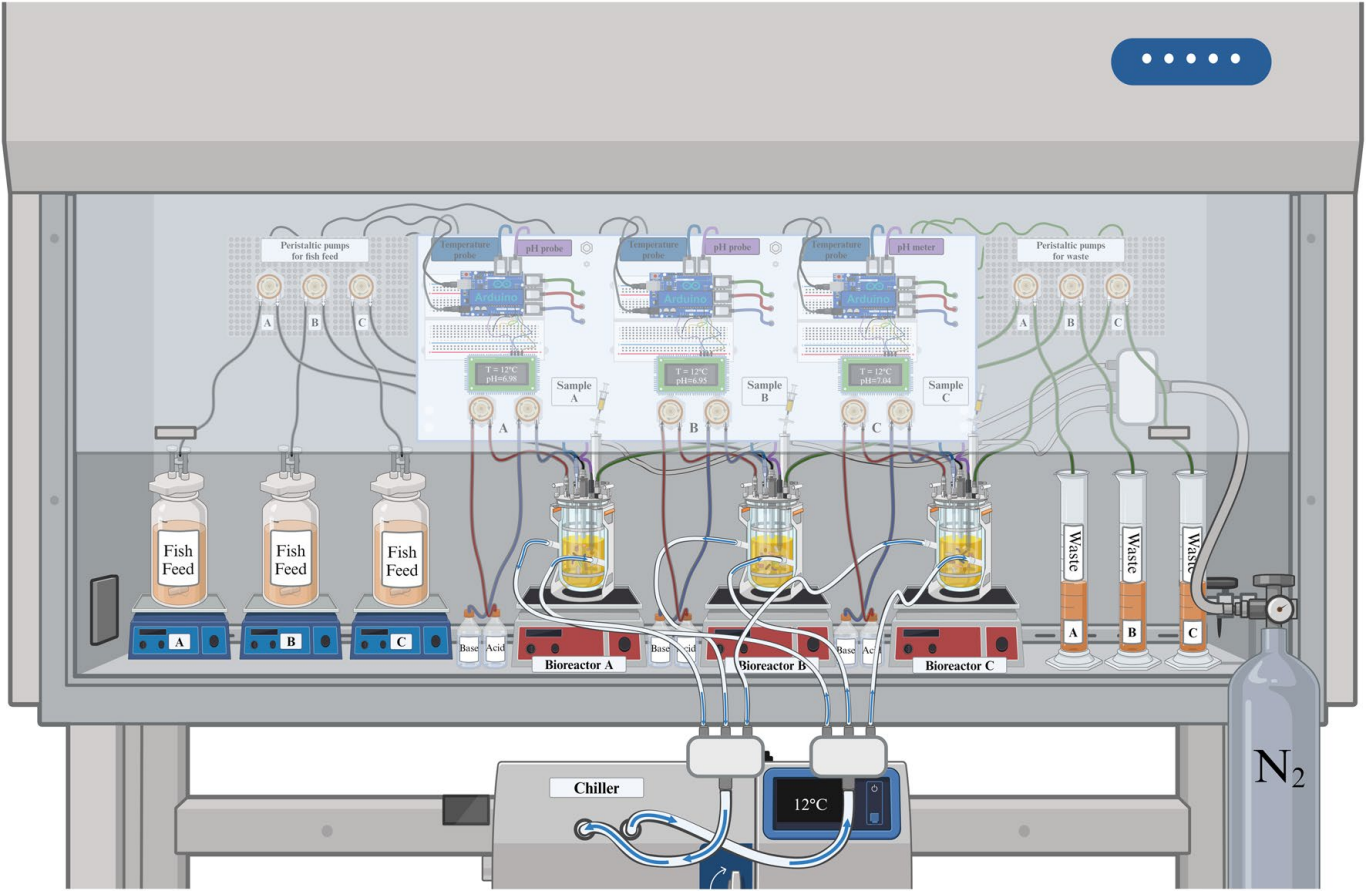
Experimental setup

In this study, we deployed the Atlantic salmon artificial gut system SalmoSim (Fig. 1) to track a multidrug-resistant (MDR) plasmid before, during and after treatment with an antibiotic widely deployed in finfish aquaculture.

Specifically, we aimed to establish (1) the host range and adaptability of the MDR pM07-1 plasmid [14] within the complex microbial community of the Atlantic salmon gut, (2) the impact of selective pressure exerted by the antibiotic florfenicol on the dynamics of plasmid transfer and the subsequent shifts in the microbial community structure, and (3) the role of different microbial taxa in promoting the persistence of AMR determinants in the absence of drug pressure.

Hi-C provides detailed spatial information about DNA interactions within microbial communities [15–18], whereas shotgun sequencing offers comprehensive genetic profiling [19, 20], allowing for the precise identification and characterization of plasmid‒host associations. Additionally, we used quantitative PCR (qPCR) to determine the plasmid concentration [21] in the bioreactors.

## Materials and methods

### Atlantic salmon gut sample collection and preparation

Three intestinal tracts were collected from healthy farmed adult Atlantic salmons. They were transported to the laboratory in an anaerobic box on ice to maintain their integrity and prevent microbial contamination. Upon arrival at the laboratory, the pyloric cecal segments were carefully separated from each intestinal sample. A total of 1 g of each gut microbial sample (comprising mucus and scrap from the internal epithelium) was extracted from each pyloric cecum and transferred into separate sterile cryovial tubes. The gut microbial samples were snap-frozen in liquid nitrogen and stored in a freezer at – 70 °C to seed the bioreactors as described below. In addition, we collected a set of “backup” samples to serve as a contingency plan to maintain the experimental integrity and allow for potential restarts, if needed.

### Fish feed medium preparation

The nutritional information of the feed pellets used for the primary in vitro culture system was previously described in [22, 23]. To prepare the feed medium, a mixture of 70 g of Instant Ocean and 20 g of ground fish pellets were combined with 2 L of Milli-Q water in a Duran bottle. To simulate the enzyme digestion of the salmon stomach, the pH of the solution was adjusted to 3.5 using hydrochloric acid (HCl), and 12 ml of crude stomach enzyme extract was added while stirring. The enzyme digestion process was carried out for one hour. The pH was subsequently restored to neutral by the addition of sodium hydroxide (NaOH). Once the pH reached 7.0, 1 g of dry sterile mucus was added to the digested media, and the entire mixture was autoclaved at 121 °C for 15 min to ensure sterility. After autoclaving, the fish meal was filtered through a sieve to remove any solid particles that could block the silicon tubes and then autoclaved once again. Each bioreactor was provided with a batch of fish feed media for the experiment.

### SalmoSim in vitro system preparation

Three 700-mL custom-made double-jacketed glass bioreactors were used in this study. To support bacterial growth, four pieces of a 1 cm^3^ aquarium sponge filter were placed in each bioreactor to enhance the three-dimensional structure. Magnetic stirrers were used for continuous mixing. The entire setup, including the bioreactors and the magnetic beads, was autoclaved to ensure sterility. A microcontroller board (Arduino Uno) was used to monitor the temperature and control and monitor the pH via automated pumping of acidic (0.1 M hydrochloric acid (HCl)) and basic (0.1 M NaOH) buffers (see Fig. 1). pH buffers and feed media were pumped via Atlas Scientific peristaltic pumps controlled by the Arduino. Initially, 400 mL of sterile feed media was added to each bioreactor. During the continuous flow phases of the experiment, fish feed was supplied at a rate of 200 ml/day, with the waste slurry removed via peristaltic pumping to maintain a constant total volume of 400 ml/bioreactor.

Figure 1 Illustration of the SalmoSim in vitro system configured within a biosafety cabinet. The system includes three 700-mL custom-made double-jacketed glass bioreactors labeled A, B, and C, each equipped with magnetic stirrers for continuous mixing and containing four pieces of 1 cm^3^ aquarium sponge filters to support bacterial growth. The temperature and pH are meticulously controlled by a microcontroller board (Arduino Uno), which regulates automated dosing of acidic and basic buffers (0.1 M hydrochloric acid and 0.1 M NaOH) via Atlas Scientific pumps to regulate the pH. Initial sterile feed media (400 mL) were introduced into each bioreactor. The feed media was continuously supplied at 200 mL/day through peristaltic pumps, while the waste was simultaneously removed to maintain a stable volume of 400 mL per bioreactor. A chiller was integrated to sustain the system at 12 °C, and a nitrogen (N_2_) tank ensured that anaerobic conditions were maintained. The detailed wiring and tubing setup depicted in the figure ensured precise monitoring and control, which is crucial for studying antimicrobial resistance in microbial communities within the artificial gut environment of Atlantic salmon. This setup facilitates a comprehensive investigation of bacterial dynamics under controlled laboratory conditions

### Inoculation and maintenance of bioreactors

Each bioreactor was inoculated with 1 g of gut contents containing intestinal microorganisms from the gut microbial sample from the pyloric cecum, which were subsequently dissolved in 1 mL of autoclaved 35 g/L Instant Ocean Sea Salt suspension. This inoculum served as the source of intestinal microorganisms, representing the microbial composition of the pyloric cecum of the fish Atlantic salmon. This step ensured the establishment of a representative microbial community in each bioreactor. The internal physiological and chemical environments of the bioreactors were carefully controlled to mimic in vivo conditions. The dissolved oxygen level in the system was minimized by sparging the bioreactors with sterile-filtered nitrogen (N_2_) gas for 20 min daily. The temperature of the bioreactors was regulated at 12 °C by pumping chilled water through the outer compartment of the double-jacketed bioreactors. To simulate the pyloric cecum compartments, the three bioreactors received daily additions of 1 mL of filtered salmon bile and 0.5 mL of autoclaved 5% mucous solution. The 5% mucous solution was prepared by mixing 5 g of mucous with 100 mL of Milli-Q water, which was then autoclaved to maintain sterility, aliquoted in 1.5 ml Eppendorf tubes and frozen at − 20 °C. This experimental setup ensured the provision of appropriate nutritional and environmental conditions, mimicking the pyloric cecum compartments of the fish. The continuous monitoring and control of pH, temperature, and nutrient addition allowed for the maintenance of a stable and representative in vitro fish gut system.

### Bacterial strains and plasmid transfer

The strain used in this study was *Escherichia coli* ATCC 29522, which is deficient in diaminopimelic acid (DAP) and harbors the MDR plasmid pM07-1 [14, 24]. To avoid the continuous use of DAP, the plasmid was transferred to *Escherichia coli* ATCC 25922 [25] via conjugation, which was performed according to the general conjugation protocol from the Barrick Laboratory [26]. This transfer step was carried out prior to the preparation of the inoculum for the subsequent experiments.

### Plasmid inoculum preparation

A florfenicol stock solution was prepared by dissolving florfenicol in dimethyl sulfoxide (DMSO) at a concentration of 50 mg/mL. The stock solution was filter sterilized in a 0.2 µm filter syringe and stored as aliquots at −20 °C.

Before the gut simulator was inoculated, ATCC 25922 harboring pM07-1 was acclimatized to the SalmoSim system conditions. Initially, it was cultured overnight in tryptic soy broth supplemented with florfenicol at a concentration of 50 μg/mL. The cultures were incubated at 37 °C with agitation at 300 rpm. To simulate the conditions of the bioreactors, customized marine media supplemented with florfenicol similar to the media used in the bioreactors were prepared. The ATCC 25922 (pM07-1) strain was transferred to customized media, which also contained florfenicol. The culture was grown overnight at 25 °C with agitation at 300 rpm. Subsequently, ATCC 25922 (pM07-1) was cultured in fish feed media supplemented with florfenicol (50 mg/L), and the culture was incubated at 14 °C for 40 h.

To prepare the inoculum, ATCC 25922 (pM07-1) grown in the fish feed media was centrifuged at 2000×*g* for 10 min. The resulting pellet was washed with sterile seawater to remove antibiotics. After washing, the pellet was suspended in 1 mL of seawater. The system was then inoculated with approximately 10^10^ colony-forming units.

### Experimental design and sampling

The experimental trial followed a predetermined timeline involving static microbial growth, fish feed supplementation, plasmid inoculation, antibiotic treatment, and a subsequent washout period. Sampling was conducted at specific time points throughout the study for analyses such as Hi-C and qPCR. Initially, the experiment involved 5 days of static microbial growth without the continuous flow of fish feed media, allowing bacterial populations to establish within the in vitro system. This was followed by a 35-day period of fish feed supplementation at 200 mL/day, which supported bacterial proliferation and activity. On day 40, the bioreactors were inoculated with plasmid-conjugated *E. coli*. Media supplementation was paused for 24 h post inoculation to allow the conjugated bacteria to settle, after which a continuous flow of feed media and waste removal resumed. This step introduces transmissible genetic material into bacterial populations to study the spread of antimicrobial resistance.

Starting on day 42, florfenicol was added to the fish feed media at 150 mg/L for 5 days, providing selective pressure to stimulate plasmid transfer between bacterial communities. This was followed by a 7-day washout period without antibiotic treatment.

**Fig. 2:**
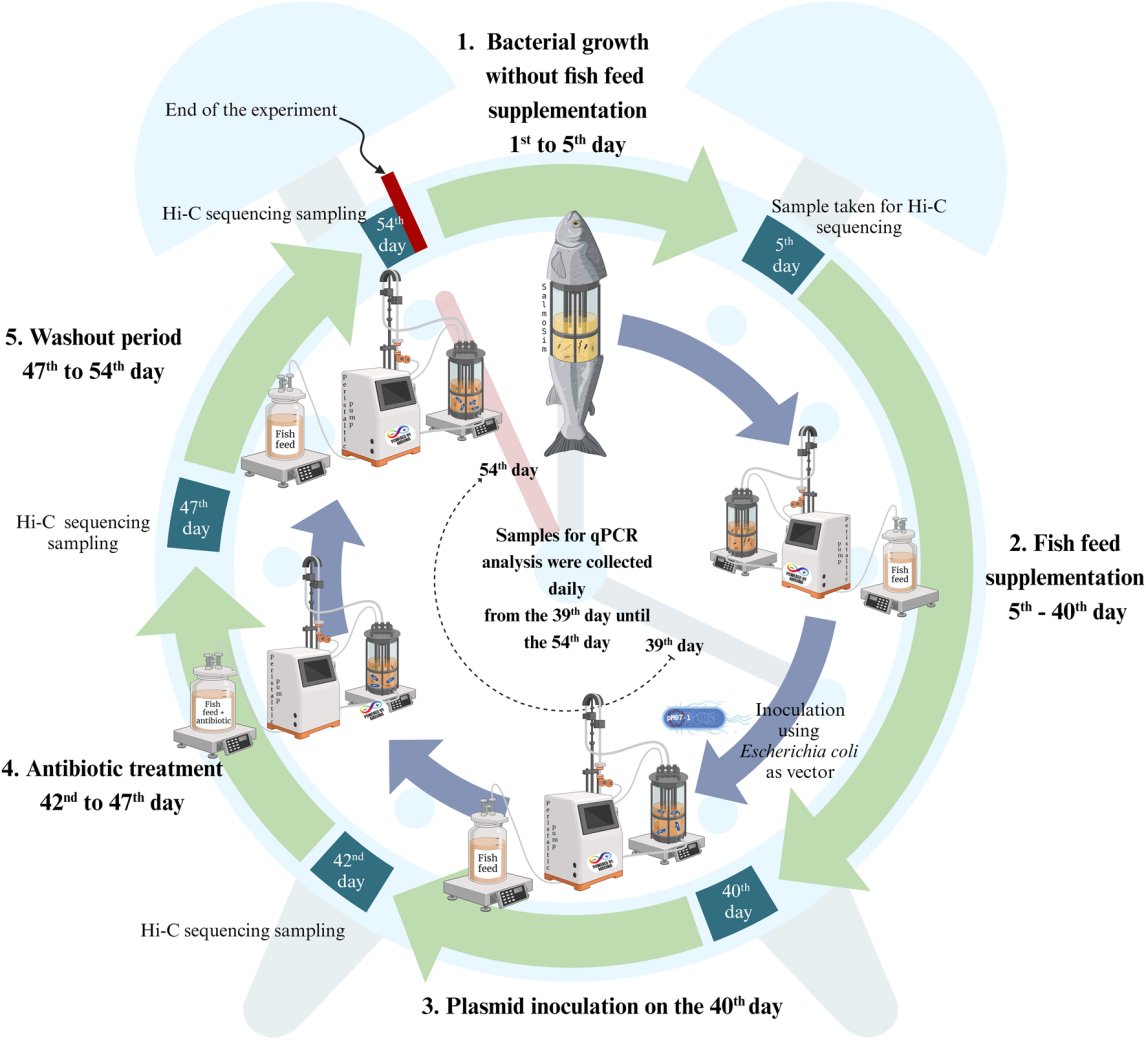
Experimental design and sampling

Samples were collected at various stages to capture different phases of the experiment. Hi-C sequencing samples were taken on day 5, and additional samples were collected on day 42 before the addition of florfenicol (pretreatment), on day 47 (the last day of antibiotic treatment; treatment phase), and on day 54 (the end of the study; washout phase). Additionally, samples for qPCR analysis were collected daily, starting on day 39 (Fig. 2).

### Analysis of the pM07-1 plasmid concentration during the main experimental trial

During the experimental trial, a 1 mL sample was collected from each sample in sterile 1.5 mL Eppendorf tubes. The collected samples were then subjected to centrifugation at 1000×*g* for 10 min at 15 °C. The supernatant was subsequently carefully removed, and the resulting pellet was suspended in 1 mL of Milli-Q water. To facilitate plasmid extraction, the samples were boiled for 15 min. After boiling, the samples were centrifuged at 13,000×*g* for 10 min to separate the supernatant. To quantify the plasmid levels in each bioreactor, quantitative real-time PCR (qPCR) was performed using 0.5 mL of the recovered supernatant obtained after centrifugation. Primers targeting the florfenicol resistance gene were used to quantify the levels of the MDR plasmid pM07-1. The specific gene associated with this plasmid was measured via qPCR with the primers flor-q-FP (GGATGGCAGGCGATATTCAT) and flor-q-RP (CTTGACTTGATCCAGAGGGC). The pM07-1 plasmid was amplified and purified via the QIAGEN Plasmid Plus Midi Kit to create standards of known concentration. This was done so that a standard curve could be generated to enable absolute quantification of experimental samples by qPCR. Serial dilutions were prepared from the purified plasmid product as follows: P1 (1 in 100), P2 (1 in 1000), P3 (1 in 10,000), P4 (1 in 100,000), P5 (1 in 1,000,000), and P6 (1 in 10,000,000). qPCR was performed via Luna® Universal qPCR Master Mix (BioLabs®, New England) at a concentration of 1x. The florfenicol resistance qPCR primers were used at a working concentration of 0.25 µM. Two microliters of either the experimental sample or the plasmid standard were used in each reaction, and the final total volume was 10 µL. Each sample was analyzed in technical duplicate. The qPCR analysis was performed in Qiagen Strip Tubes in a Qiagen qPCR machine (Rotogene Q, Hilden, Germany).

The plasmid copy number in each sample was calculated by interpolating the Ct values from the bioreactor samples onto the standard curve equation. The copy number was expressed as copies/μl of the sample. The standard curve equation used for this calculation was derived from a linear regression of the known plasmid concentrations and their corresponding Ct values via the following equation:

$${\text{Log}}\;\left( {{\text{copy}}\;{\text{number}}} \right) \, = {\text{ a}} \times \left( {{\text{Ct}}} \right) + {\text{b}},$$

where a and b are the slope and intercept of the standard curve, respectively. This equation allows for the determination of plasmid copy numbers in the experimental samples by substituting the Ct values into the equation and solving for the copy number.

### Sample preparation for Hi-C sequencing

**Fig. 3:**
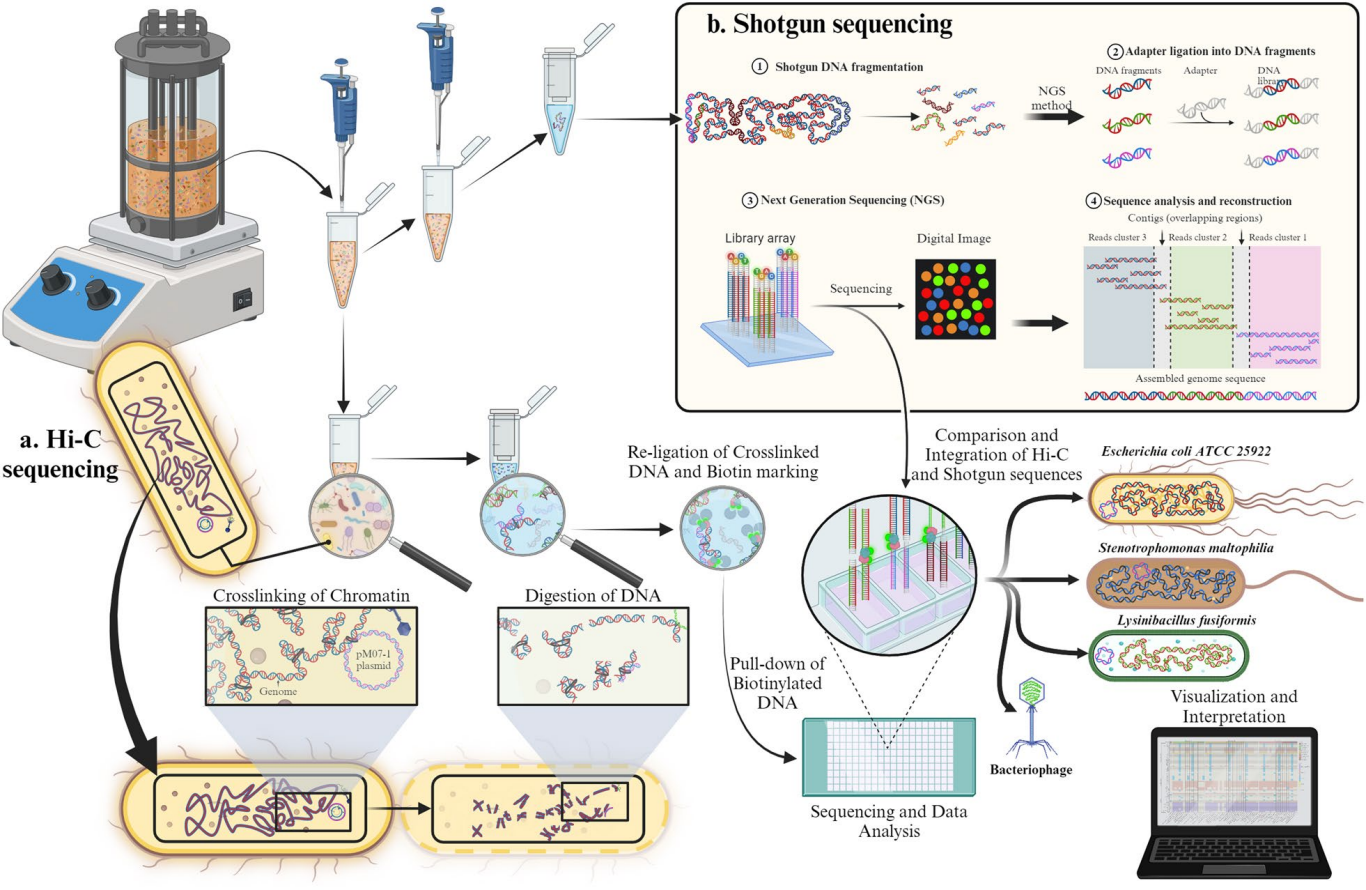
Integrated Hi-C and shotgun sequencing analysis of microbial samples from bioreactors

A 10 mL sample from each bioreactor was collected on days 5, 42, 47, and 56 in 15 mL sterile tubes and centrifuged at 3000×*g* for 10 min. The supernatant was discarded, and the pellet was resuspended in 0.5 mL of Phase Genomics PGShield™. The samples were subsequently sent to Phase Genomics (Seattle, WA, U.S.A.) for further analysis (see Fig. 3).

### ProxiMeta methods

A Hi-C library was created with the Phase Genomics ProxiMeta Hi-C v4.0 Kit following the manufacturer-provided protocol [27]. Briefly, intact cells were crosslinked via a formaldehyde solution, simultaneously digested via the Sau3AI and MlucI restriction enzymes, and proximity ligated with biotinylated nucleotides to create chimeric molecules composed of fragments from different regions of genomes that were physically proximal in vivo. Proximity-ligated DNA molecules were pulled down with streptavidin beads and processed into an Illumina-compatible sequencing library. Separately, using an aliquot of the original sample, DNA was extracted with a ZYMObiomics DNA miniprep kit [28], and a metagenomic shotgun library was prepared via ProxiMeta library preparation reagents. Sequencing was performed on an Illumina NovaSeq platform, generating PE150 read pairs for both the Hi-C and shotgun libraries. Hi-C and shotgun metagenomic sequencing files were uploaded to the Phase Genomics cloud-based bioinformatics portal for subsequent analysis (see Fig. 3a).

Shotgun reads were filtered and trimmed for quality, normalized via fastp v0.20.1 [29] and then assembled via MEGAHIT v1.2.9 [30, 31] via default options (Fig. 3b). Hi-C reads were then aligned to the assembly following the Hi-C kit manufacturer's recommendations [32]. Briefly, reads were aligned via BWA-MEM v0.7.17 [33] with the -5SP options specified and all other options default. SAMBLASTER v0.1.26 [34] was used to flag PCR duplicates, which were subsequently excluded from the analysis. The alignments were then filtered with SAMtools v1.11 [35] via the -F 2304 filtering flag to remove nonprimary and secondary alignments. Metagenome deconvolution was performed with ProxiMeta [36, 37], resulting in the creation of putative genome and genome fragment clusters. Clusters were assessed for quality via CheckM v1.1.3 [38] and assigned preliminary taxonomic classifications via Mash v2.2 [39].

As shown in Fig. 3a, the Hi-C sequencing process involves crosslinking chromatin within bacterial cells, followed by digestion of the DNA and religation of the crosslinked fragments, which are then biotin labeled. These labeled fragments are pulled down and sequenced, providing spatial information about DNA interactions within the cell. Simultaneously, as shown in Fig. 3b, shotgun sequencing involves fragmenting DNA, ligating adapters, and using next-generation sequencing (NGS) to generate a library of sequences. This method allows for the comprehensive analysis and reconstruction of the bacterial genome. The integration of Hi-C and shotgun sequencing data enables a detailed comparison and comprehensive understanding of the genetic structure and interactions of the microbial community. The final visualization and interpretation of the data reveal insights into the genomic organization and potential antimicrobial resistance mechanisms in bacterial species such as *Escherichia coli*, *Stenotrophomonas maltophilia*, and *Lysinibacillus fusiformis*.

### Taxonomic profiling

For the contigs generated from phase genomics, taxonomic profiling of the microbial communities present was conducted via Kraken version 2.1.2, which leverages a specialized microbial database to increase the accuracy and specificity of the identification process. The subsequent estimation of species abundance was performed with Bracken, which applies a threshold parameter of 100 (-r) and is configured to identify taxa exclusively at the genus level.

Following the taxonomic profiling, the microbial abundance data for each sample were visualized in RStudio. This involved the use of packages such as tidyr, dplyr, and ggplot2. To facilitate the calculation of abundances, the data were transformed to a long format. The top 25 taxa were identified by summing their abundances, and these taxa, along with an aggregated "Others" category, were plotted to highlight their relative proportions.

### Whole-genome sequencing, contig assembly, and annotation

The *Escherichia coli* strain ATCC 25922 was isolated from the bioreactor inoculum, and its DNA was extracted via the Qiagen REPLI-g single-cell kit following the manufacturer's instructions. The extracted DNA was sent to GENEWHIZ for whole-genome sequencing (Illumina NovaSeq). Genome assembly was conducted via SPAdes 4.0.0 [40]. Annotation of antimicrobial resistance genes was performed via the Comprehensive Antimicrobial Resistance Database (CARD) [41] through the PROKSEE server [https://beta.proksee.ca/projects]. The PROKSEE server also generated high-quality, navigable maps for the AMR genes of the whole genome, as well as for the plasmid PM07-1 and its AMR genes.

## Results

### Variations in microbial community dynamics across treatment stages and bioreactors

**Fig. 4:**
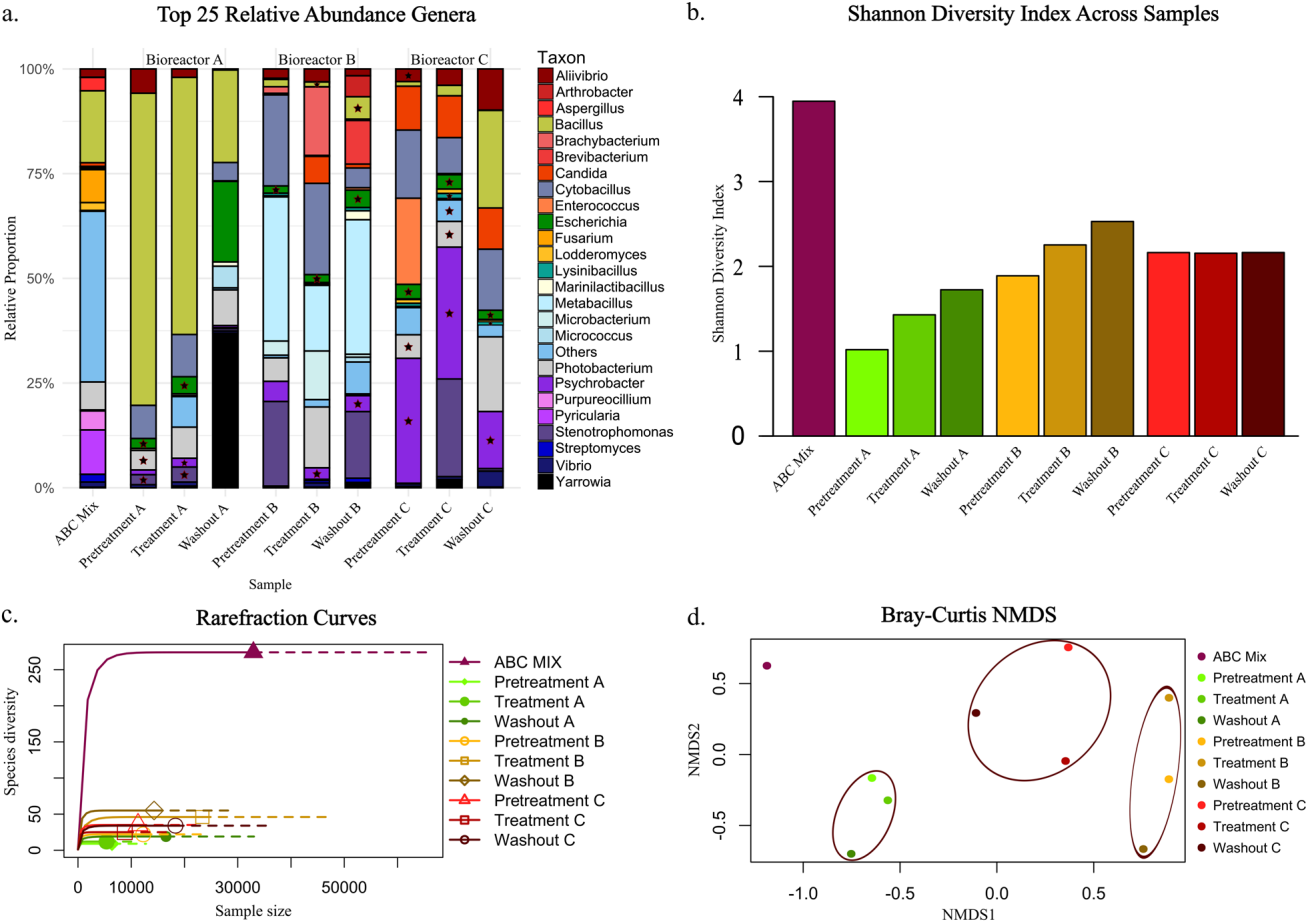
Species diversity and relative abundance of the top 25 genera across all the samples. **a** Variation in the relative abundance of the top 25 taxa across samples in different bioreactor phases. The ABC mix bar represents a composite from bioreactors A, B, and C after 5 days of static growth prior to the initiation of continuous flow with fish feed. Stars indicate genera with the florfenicol resistance gene. Diverse, less abundant genera are represented as "Others." **b** Shannon diversity index. The Y-axis represents the Shannon diversity index, which ranges from 0 to 4. **c** The rarefaction curves show that the ABC mixture sample, which has the highest genus diversity, plateaus at 250 taxa. The other samples presented lower diversity, were less than 50 species, and presented less pronounced plateaus, indicating lower richness. **d** The Bray‒Curtis NMDS plot highlights relationships between microbial community compositions across bioreactor samples. Brown ellipses encapsulate clusters, showing samples from the same bioreactor. The ABC mixture (dark purple) is distinct, indicating a unique microbial community.

The analysis of microbial communities revealed notable variations in genus abundance and diversity across different treatment stages and bioreactors, indicating that dynamic yet moderate changes were influenced by the treatment process. The relative proportions of genera varied significantly across the samples in different phases within each bioreactor (Fig. 4a), and several genera dominated specific samples. A detailed comparison of the similarities and differences in identified genera across bioreactors A, B, and C is provided in Supplementary Table S1. This summary highlights the presence and relative abundance of key genera across experimental phases, facilitating a better understanding of microbial interactions and their potential influence on AMR transfer.

The ABC mixture sample was a composite of samples taken from three separate bioreactors, labeled A, B, and C, after a period of 5 days of static growth prior to the initiation of continuous flow with fish feed. AB&C were pooled in this way to provide a broad overview of the initial microbial community established across bioreactors in the experiment. The high diversity observed in the ABC mixture sample, as demonstrated by the Shannon diversity plot (Fig. 4b) and the rarefaction curve plot (Fig. 4c), indicated that a substantial portion of the microbial community comprised a wide array of genera that were not individually dominant enough to be included in the top 25 most abundant taxa. Consequently, these less abundant but diverse genera are collectively represented as "Others" in Fig. 4a, reflecting the rich microbial diversity present in the ABC Mix sample.

In bioreactor A, there was a marked decrease in the populations of *Bacillus* and *Aliivibrio* across the experimental phases, accompanied by an increase in the populations of *Escherichia* and *Stenotrophomonas*, both of which possess the florfenicol resistance gene. Similarly, Bioreactor B exhibited an increase in populations of bacterial taxa with the florfenicol resistance gene during the treatment phase, including *Bacillus* and *Psychrobacter*. In bioreactor C, the population of the dominant group, *Psychrobacter*, decreased during the washout phase, whereas the population of *Bacillus* increased (Fig. 4a).

Figure 4b shows that the Shannon diversity index, which measures the richness and evenness of microbial communities, was highest in the ABC mixture sample. However, after 37 days of continuous flow with fish feed, a significant decrease in Shannon diversity was observed across all bioreactors (A, B, and C) compared with the ABC mixture sample. Interestingly, bioreactors A and B exhibited noticeable increases in diversity during the three experimental stages: pretreatment, treatment, and washout. In contrast, bioreactor C maintained a relatively constant level of Shannon diversity across all experimental stages, indicating a stable and consistent microbial community composition throughout the pretreatment, treatment, and washout phases of the study.

In Fig. 4c, the rarefaction curves depict the species diversity as a function of sample size. ABC MIX shows the highest diversity, reaching a plateau of approximately 250 species. Compared with the ABC MIX treatment, the other treatments and washouts resulted in markedly lower species diversity, generally below 50 species, with much less pronounced plateaus, indicating lower species richness. The Bray‒Curtis NMDS plot (Fig. 4d) visually demonstrates the differences and similarities in microbial community compositions across samples from different bioreactors and stages. The ABC mixture (dark purple) is distinct from the other samples, indicating a divergent microbial community. The samples from bioreactors A (light green, green, and dark green), B (yellow, orange, and brown), and C (light red, red, and dark red) clustered together in the bioreactor, suggesting similar community compositions within each bioreactor across stages but were distinct between the bioreactors. This finding indicates that microbial communities are more similar within each bioreactor throughout stages and that each bioreactor hosts a diver, relatively consistent community composition.

During the washout phase of Bioreactor A, we observed a noticeable increase in *Yarrowia* populations (Fig. 4a). A mechanical failure of the magnetic stirrer responsible for maintaining bioreactor homogenization, which occurred 5 days before the last sampling point, likely underpins this divergent profile. Specifically, the magnetic stirrer maintaining the homogenization of the bioreactor stopped working overnight, causing the internal magnet to cease spinning. This malfunction resulted in inadequate mixing within the bioreactor. Consequently, the pH probe detected a slightly acidic environment and instructed the Arduino system to pump more sodium chloride to adjust the pH to 7. However, due to insufficient mixing, the pH in the bioreactor increased significantly, exceeding the probe's readable range. Although the issue was promptly corrected the following morning, the extreme pH conditions likely led to a substantial reduction in most bacterial populations, providing an opportunity for the *Yarrowia* group to thrive under these altered conditions.

### Distribution and AMR gene transfer of the plasmid pM07-1 across bioreactors

To investigate the distribution and horizontal gene transfer of the plasmid pM07-1, which carries multiple antimicrobial resistance genes, we conducted a series of experiments in which bioreactors A, B, and C were inoculated with the *Escherichia coli* strain ATCC 25922. Additionally, we monitored plasmid concentrations and the distribution of AMR genes across the pretreatment, treatment, and washout stages.

**Fig. 5:**
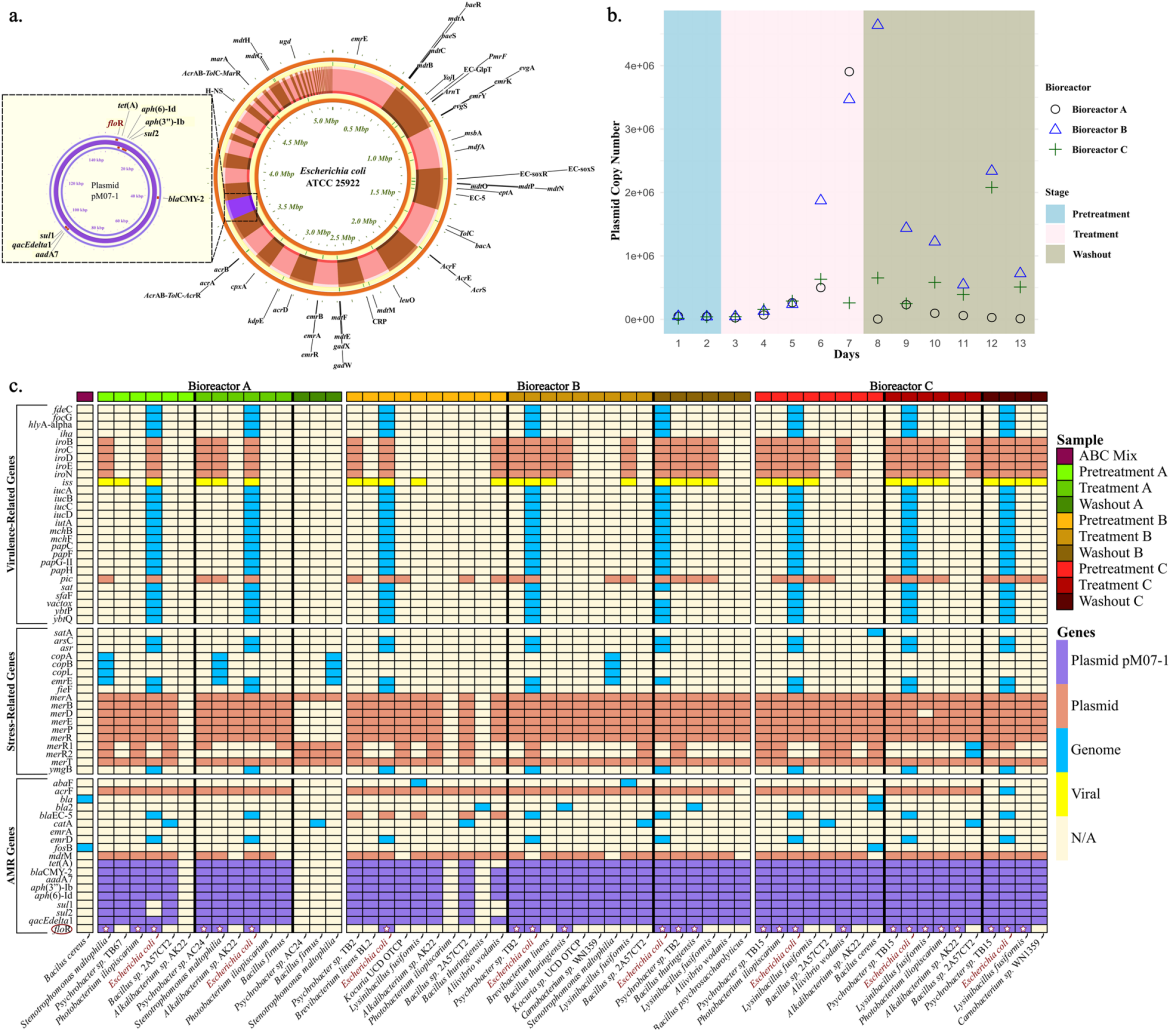
Plasmid distribution, concentration dynamics, and gene prevalence across all samples. **a**. Circular genome map of the *Escherichia coli* strain ATCC 25922 with the plasmid pM07-1, highlighting nine AMR genes, including *flo*R, *tet*(A), *APH*(6)-Id, *sul*2, CMY-2, *aad*A7, and *qac*E*delta*1, which confer resistance to multiple antibiotics. **b**. Plasmid copy numbers over 13 days in bioreactors A, B, and C during the pretreatment, treatment, and washout stages. **c**. Heatmap showing AMR, stress-related, and virulence genes across bioreactor samples A (green), B (brown), and C (red) during the pretreatment, treatment, and washout stages. The plasmid pM07-1 is shown in purple. *Escherichia coli*, the plasmid vector used, is highlighted in red

Our results demonstrated that the genome of the inoculum bacteria, *E. coli* ATCC 25922, contained 58 AMR genes when analyzed via the CARD database, with a threshold for the best identity contig-AMR match exceeding 90%. In particular, nine of these AMR genes were located on the plasmid pM07-1, which is approximately 150 kb in size (Fig. 5a). The complete genome assembly of *E. coli* ATCC 25922 was previously published and submitted to NCBI under accession number CP009072 [25]. This strain, which was isolated from a clinical sample in Seattle, Washington (1946), and is commonly used in quality control testing, originally included two plasmids. However, after introducing plasmid pM07-1 through a conjugation process from an *E. coli* K12 strain deficient in diaminopimelic acid and subsequently isolating bacteria from the bioreactor inoculum, we discovered the presence of three plasmids. These genes confer resistance to various antibiotics, including *flo*R (chloramphenicol and florfenicol resistance), *tet*(A) (tetracycline resistance), *APH*(6)-Id and *APH*(3'')-Ib (aminoglycoside resistance), *sul*2 and *sul*1 (sulfonamide resistance), CMY-2 (carbapenem, cephalosporin, and penicillin resistance), *aad*A7 (spectinomycin and streptomycin resistance), and *qacEdelta*1 (resistance to disinfecting agents and antiseptics).

The results of the qPCR analysis of the florfenicol gene (Fig. 5b) revealed that during the pretreatment stage (Days 1–2), the plasmid concentrations in all three bioreactors (A, B, and C) remained low and consistent. During the treatment stage (Days 3–7), a notable increase in plasmid concentration was observed in bioreactor B starting from Day 6, when it reached approximately 2 million copies/μl by Day 7. Bioreactor A also showed an increase in plasmid concentration, peaking on Day 7, although the increase was smaller than that in bioreactor B. Bioreactor C also presented an increase in plasmid concentration, but it was less pronounced than that in bioreactor B. In the washout stage (Days 8–13), plasmid concentrations in bioreactor B continued to rise, peaking at approximately 4 million copies/μl on Day 9, and fluctuated between 2–4 million copies/μl until Day 13. In contrast, bioreactor A resulted in a decrease in the plasmid concentration after day 7, with low values by day 13. Bioreactor C displayed a modest increase in plasmid concentration, peaking around Day 11 and maintaining a higher concentration than did bioreactor A but lower than that of bioreactor B. Antibiotic selective pressure during the last 2 days of the treatment phase significantly increased plasmid abundance across all bioreactors. Specifically, plasmid abundance increased significantly in all observations during the treatment phase compared with the pretreatment phase (*p* < 0.05, calculated via a paired t test). Furthermore, there was no significant decrease in plasmid abundance during the washout phase, with 2 out of 3 observations showing no significant change compared with the treatment phase (p > 0.05, paired t test).

Figure 5c shows the distribution and transfer of AMR genes associated with the plasmid pM07-1 across various bioreactor samples, which are highlighted in purple at the bottom of the heatmap. We identified plasmid transfer to 17 different bacterial species, comprising 7 g-negative and 10 g-positive species (Fig. 5c).

Successful plasmid pM07-1 transfer was defined as the presence of at least 8 out of 9 antibiotic resistance genes encoded on the plasmid in the recipient bacterial community. This threshold was established for potential minor variations in gene transfer efficiency and sensitivity of detection, which might cause the occasional absence of a single gene.

In the ABC mixture sample, only two AMR genes, *bla* and *fos*B, are located on the bacterial chromosome of *Bacillus cereus*. *Escherichia coli*, the vector for the plasmid, consistently expresses the plasmid-specific AMR genes at all stages across the bioreactors, except in the ABC mix.

In Bioreactor A, during the pretreatment phase, *Photobacterium iliopiscarium* and *Stenotrophomonas maltophilia* acquired the plasmid pM07-1. Additionally, successful plasmid acquisition occurred in *Psychrobacter* sp. TB67 and *Bacillus* sp*.* 2A57CT2*.* During the treatment phase, these same species continued to harbor the plasmid, with the addition of *Alcalibacterium* sp. AK22. However, in the washout stage, no plasmid was observed, likely because of the disruption caused by the magnetic stirrer described previously.

In the pretreatment stage of bioreactor B, the plasmid was detected in seven species, indicating extensive horizontal gene transfer. This phase also resulted in greater plasmid-associated AMR gene biodiversity, with new species such as *Brevibacterium linens* BL2, *Kocuria* UCD OTCP, and *LysiniBacillus fusiformis*, along with *Aliivibrio wodanis* showing the presence of the plasmid pM07-1. In the treatment phase, plasmid transfer increased with the addition of *Bacillus thuringiensis*, which acquired the plasmid despite not having it during pretreatment. We also identified the plasmid in six other species. During the washout phase, the plasmid stably persisted in five species, including *Psychrobacter* sp. TB2 and *Bacillus thuringiensis*, along with three other species that still carried the plasmid.

In Bioreactor C, during the pretreatment phase, the plasmid pM07-1 was detected in seven species. Interestingly, during the treatment phase, the presence of the plasmid was observed in only five species. In the washout phase, the plasmid persisted in three species.

## Discussion

In this study, we provide the first survey of the endogenous AMR complement in the gut microbiota of the Scottish *Salmo salar*. Using Hi-C technology coupled with shotgun sequencing, we then established a direct link between the MDR plasmid pM07-1 and its microbial hosts. This approach enables detailed mapping of the presence and distribution of AMR genes, providing new insights into microbial ecology and resistance dynamics within the aquaculture environment. Here, we show that the pM07-1 plasmid interacts with numerous bacterial hosts spanning gram-negative and gram-positive taxa and, in particular, continues to be transferred and persists once florfenicol treatment has been removed, emphasizing the role of the commensal teleost gut flora as a reservoir for AMR.

The SalmoSim in vitro simulation platform provides a transformative framework for investigating microbial ecology and AMR in aquaculture systems. Unlike in vivo models restricted by ethical considerations, biological variability, and the logistical challenges of monitoring dynamic microbial communities, SalmoSim enables precise control over environmental parameters [22, 23]. This approach permits detailed interrogation of plasmid transfer and microbial interactions, free from the welfare constraints of animal-based research. Moreover, in contrast to ex vivo methodologies, SalmoSim supports continuous nutrient supplementation under controlled anaerobic and temperature-regulated conditions. The resulting environment closely approximates the teleost gut, offering a more accurate and ethically sustainable surrogate for in vivo studies*.*

We noticed a substantial shift in the microbial communities across the different experimental phases. Notably, the ABC mixture sample presented the highest microbial diversity, as evidenced by the rich variety of genera represented. This initial high diversity could be attributed to the fact that the sample represents a pool of all the bioreactors early in the experiment. Additionally, as we noted previously in SalmoSim, rare microbial taxa are progressively lost as the community adapts to culture conditions [22]

As the bioreactors transitioned to continuous flow with fish feed, a marked decrease in microbial diversity was observed. This is supported by the Shannon diversity index (Fig. 4 Panel b) and rarefaction curves (Fig. 4 Panel c), both of which indicate significant reductions in species richness and evenness. We used florfenicol as a stress factor in our study because it is commonly used in aquaculture to treat bacterial infections [42, 43], making it relevant for simulating real-world conditions and stress factors. The continuous flow environment likely imposed selective pressures that favored certain genera capable of thriving under these conditions, leading to a reduction in overall diversity, since florfenicol has broad-spectrum antibacterial properties that are effective against a wide range of pathogens, including those resistant to other antibiotics [44]. In aquaculture, florfenicol is typically administered via medicated feed at doses of 10–15 mg/kg fish/day for approximately 10 consecutive days, as recommended by regulatory guidelines and product bulletins [45, 46]. Here, we employed a substantially higher concentration (150 mg/L) of florfenicol within the SalmoSim in vitro system to impose a pronounced selective pressure on the microbial community. This concentration surpasses typical in vivo exposures, and is designed to simulate worst-case scenarios such as local drug accumulation or accidental overdosing.

In bioreactor A, a decrease in the *Bacillus* and *Aliivibrio* populations was accompanied by an increase in the *Escherichia* and *Stenotrophomonas populations*, both of which carry florfenicol resistance genes. These findings suggest that the selective pressure exerted by florfenicol may have facilitated the proliferation of resistance genera. Similarly, Bioreactor B presented an increase in *Bacillus* and *Psychrobacter* populations, both of which are associated with florfenicol resistance during the treatment phase.

The changes observed in Bioreactor C were distinct, with *Psychrobacter* initially dominant but decreasing during the washout phase, whereas *Bacillus* populations increased. This variation indicates that different bioreactors may harbor unique microbial dynamics and resistance patterns, influenced by the founding community composition and subsequent environmental conditions [47].

Interestingly, while bioreactors A and B exhibited fluctuations in diversity across the experimental stages, bioreactor C maintained a relatively stable microbial community composition. This stability, as shown by the consistent Shannon diversity index across the pretreatment, treatment, and washout phases, suggests that certain microbial communities can achieve equilibrium despite the introduction of selective pressures [48]. The stability of bioreactor C might be attributed to the initial community's resilience or adaptive mechanisms that buffer against environmental changes.

As shown in Fig. 4d, the clear clustering of samples by bioreactor indicates that microbial communities within each bioreactor are more similar to each other across stages than to those in other bioreactors. Importantly, each of the three bioreactors represented microbial communities derived from different individual Atlantic salmon. The significant differences observed in the bacterial community composition and diversity dynamics across these bioreactors likely reflect the inherent individual variations among the host fish. These individual differences could arise from factors such as genetic background, early-life environmental exposures, and potentially distinct host‒microbe interactions [49].

Furthermore, the identification of nine AMR genes on the plasmid, including the florfenicol resistance gene, underscores the significant role that plasmids play in harboring and disseminating resistance traits. The presence of these genes, which confer resistance to various antibiotics, poses a substantial threat to the efficacy of antimicrobial treatments.

Plasmid transfer from gram-negative to gram-positive bacteria is rare and typically involves a shuttle, a type of plasmid that has the ability to replicate in multiple host species [50, 51], or broad host range plasmids such as IncP [52] and IncPromA [53], with narrower host plasmids such as IncX3 also showing cross‒gram transfer [54]. While previous studies have reported intergram plasmid transfer for other plasmid groups, our findings demonstrate that the MDR IncA/C plasmid pM07-1 can also traverse the gram-negative/gram-positive boundary.

Here, we detected plasmid transfer in 17 distinct bacterial species, including seven gram-negative strains and ten gram-positive strains (Fig. 5c). These species belong to seven different bacterial families, demonstrating the broad host range and adaptability of the plasmid. The gram-negative species included members of the families *Vibrionaceae*, *Moraxellaceae*, and *Xanthomonadaceae*, whereas the gram-positive species were distributed among the families *Bacillaceae*, *Carnobacteriaceae*, *Brevibacteriaceae*, and *Micrococcaceae*.

There are many probiotics on the market that are *Bacillus*-based formulations, and one major concern regarding the use of probiotics is that they can potentially acquire AMR genes through mobile genetic elements. This poses a major risk in animal production systems, where such transfer could lead to the spread of resistance traits, complicating efforts to control infections and maintain animal health [55].

The presence of the resistance gene *bla*CMY-2 for carbapenems, cephalosporins, and penicillins is particularly alarming [56]. These classes of antibiotics are among the most effective treatments currently available for combating pathogenic bacteria, including MDR strains [57]. The ability of the pM07-1 plasmid to confer resistance to these critical antibiotics indicates that the plasmid has the potential to significantly compromise treatment options for severe bacterial infections.

During the washout phase of bioreactor A, none of the nine AMR genes associated with plasmid pM07-1 were detected. The extremely high pH in bioreactor A likely caused notable bacterial death, including that of the plasmid pM07-1 carriers, resulting in a drastic decrease in plasmid abundance. Additionally, surviving bacteria may have lost the plasmid due to stress-induced metabolic burdens [58]. Consequently, no plasmids or associated AMR genes were detected during the washout phase, indicating plasmid instability under harsh conditions. [59]. In addition, there was a notable shift in the microbial community structure. Specifically, the relative abundance plot revealed a significant increase in the *Yarrowia* population. The elevated pH conditions likely inhibited other highly abundant bacteria, creating an opportunity for *Yarrowia* to increase in population. As a yeast commonly used in fish feed as a probiotic [60, 61], *Yarrowia* can thrive in altered environments [62, 63], taking advantage of the reduced competition from other microorganisms that are less tolerant to high pH conditions.

Hi-C is a powerful tool for use in metagenomics studies; however, it has some limitations that can affect data interpretation. The observed inconsistencies in the presence of certain AMR genes (e.g., *flo*R, *sul*1, *sul*2) across different bacterial species, despite all nine genes being present on plasmid pM07-1, can be attributed to several factors related to the Hi-C method used in our study. Insufficient sequencing depth or challenges in mapping short reads accurately to repetitive plasmid sequences can lead to missing gene signals [64]. Additionally, the Hi-C method can introduce biases in the data, such as preferential capture of certain regions over others, affecting the consistency of gene detection across samples [65]. Furthermore, limitations in the resolution and coverage of Hi-C sequencing may result in incomplete detection of plasmid sequences in some bacterial species or samples, leading to discrepancies in the observed gene signals [66].

A stable baseline prior to selective pressure application is indicated in panel B (Fig. 5b), and despite the low plasmid concentrations observed during the pretreatment phase, we can clearly observe from panel C of Fig. 5 that notable HGT occurred even at this stage. The presence of antimicrobial resistance genes across multiple bacterial species indicates that the plasmid pM07-1 actively transferred resistance traits among the microbial community. This early HGT activity suggests that even minimal plasmid presence can facilitate the spread of resistance genes, highlighting the persistent and pervasive nature of HGT in microbial ecosystems. The pronounced increase in plasmid concentration in bioreactor B during the treatment and washout stages aligns with the high diversity of AMR genes and the introduction of new species harboring these genes. These findings suggest that the selective pressure from antibiotics not only increased the plasmid concentration but also facilitated the active transfer and establishment of the plasmid in novel bacterial hosts.

The first sample taken for Hi-C was a mixture from all three bioreactors to identify the AMR genes in the intestinal flora of the three Atlantic salmon used in the experiment before the addition of the fish feed and the MDR plasmid. We discovered only two AMR genes, *bla* and *fos*B, located on the chromosome of *Bacillus cereus* (Fig. 5c). These genes are significant because they confer resistance to antibiotics commonly used in human medicine, with *bla* encoding beta-lactamase enzymes that provide resistance to beta-lactam antibiotics [67] and *fos*B conferring resistance to fosfomycin [68], posing a potential threat to the spread of AMR. Although these genes are not plasmid-borne, they still present a risk owing to the potential for horizontal gene transfer through transformation [69], transduction [70], and conjugation of chromosomal genes [71].

The low incidence of AMR genes in the first sample, the ABC mixture, is consistent with the minimal use of antibiotics in Scottish aquaculture, where antibiotic consumption has substantially decreased in recent years [72–74]. Our findings' minimal variety of AMR genes most likely reflects the efficiency of these interventions, since the sector has shifted to more sustainable methods, lessening the selective pressure for the emergence and spread of antibiotic resistance.

The presence of pM07-1 in multiple species, including *Photobacterium iliopiscarium* and *Stenotrophomonas maltophilia*, indicates efficient plasmid transfer and stable maintenance across diverse bacterial hosts. *Stenotrophomonas maltophilia* is a known human pathogen that is particularly prevalent in hospital environments, where it causes various infections, especially in immunocompromised patients. Notably, the *bla*CMY-2 gene on plasmid pM07-1, which provides resistance to multiple β-lactam antibiotics, underscores the potential for cross-species transmission of resistance traits and the associated zoonotic risk. The historical context of this plasmid, which originated from the catfish pathogen *Edwardsiella ictaluri* [14], further highlights the interconnectedness of antimicrobial resistance across different species and environments.

Interestingly, we observed a notable pattern in the bacterial taxa associated with plasmid pM07-1 across the bioreactors. With the exception of *Kocuria* UCD, every other bacterium carrying the plasmid was found in at least two different bioreactors. These findings suggest the potential resilience and adaptability of these taxa in the acquisition and maintenance of plasmids across various environmental conditions within bioreactors. The recurrence of these bacterial taxa in multiple bioreactors indicates that certain species may be more predisposed to plasmid acquisition and retention, highlighting their potential role in plasmid dissemination within microbial communities.

The findings from this study have profound implications for antimicrobial resistance management in aquaculture and other environments where microbial communities are exposed to antibiotics. The rapid and extensive HGT of resistance genes highlights the need for stringent monitoring and management practices to prevent the spread of MDR bacteria.

Understanding the dynamics of plasmid transfer and the conditions that favor the maintenance and dissemination of resistance genes can inform the development of strategies to mitigate the spread of antimicrobial resistance. This knowledge is crucial for designing interventions that target key points in the microbial community's response to selective pressures, potentially curbing the proliferation of resistant strains.

Future research should focus on in-depth mechanistic studies of HGT and plasmid stability under diverse environmental conditions. Investigating the specific factors that enhance or inhibit plasmid transfer and maintenance can provide valuable insights into controlling the spread of resistance genes. While this study presents notable strengths, several limitations must be noted. First, while Hi-C sequencing is a powerful tool, it can introduce potential biases, such as preferentially capturing certain genomic regions and failing to fully detect AMR genes present on plasmids. Second, although the controlled conditions of the SalmoSim system allow for the isolation of specific variables, they do not fully capture the complexity of natural aquaculture environments, where microbial communities are shaped by a range of ecological factors. Moreover, the chosen florfenicol concentration (150 mg/L) surpasses typical field concentrations, thus amplifying the observed AMR signal and potentially overestimating plasmid transfer rates relative to real-world scenarios. Future research should replicate these findings in field settings and investigate AMR transfer dynamics at lower antibiotic concentrations to strengthen ecological validity.

## Conclusions

In summary, our findings revealed that (1) plasmid pM07-1 has a broad host range, transferring to 16 bacterial species of both gram-negative and gram-positive bacteria, and (2) under florfenicol selective pressure, plasmid transfer increased, leading to an increase in plasmid concentrations and shifts in the microbial community structure, favoring resistant strains while reducing diversity among susceptible populations. (3) Plasmid transfer and maintenance seem to have a low cost, can occur in the absence of selection pressure, and can persist in a wide variety of microbial taxa in the absence of selection.

## Supplementary Information

Additional file 1.

## Data Availability

The data supporting the findings of this study have been deposited in the NCBI Sequence Read Archive (SRA) under BioProject accession number PRJNA1135464.

## References

[1] Christaki E, Marcou M, Tofarides A. Antimicrobial resistance in bacteria: mechanisms, evolution, and persistence. J Mol Evol. 2020;88(1):26–40. 31659373 10.1007/s00239-019-09914-3

[2] Ikhimiukor OO, Odih EE, Donado-Godoy P, Okeke IN. A bottom-up view of antimicrobial resistance transmission in developing countries. Nat Microbiol. 2022;7(6):757–65. 35637328 10.1038/s41564-022-01124-w

[3] Despotovic M, de Nies L, Busi SB, Wilmes P. Reservoirs of antimicrobial resistance in the context of one health. Curr Opin Microbiol. 2023;73:102291. 36913905 10.1016/j.mib.2023.102291 PMC10265130

[4] Klümper U, Recker M, Zhang L, Yin X, Zhang T, Buckling A, Gaze WH. Selection for antimicrobial resistance is reduced when embedded in a natural microbial community. ISME J. 2019;13(12):2927–37. 31384011 10.1038/s41396-019-0483-z PMC6864104

[5] Shepherd MJ, Fu T, Harrington NE, Kottara A, Cagney K, Chalmers JD, Paterson S, Fothergill JL, Brockhurst MA. Ecological and evolutionary mechanisms driving within-patient emergence of antimicrobial resistance. Nat Rev Microbiol. 2024;22:650–665. 10.1038/s41579-024-01041-1 38689039

[6] Shapiro JT, Zorea A, Kav AB, Ontiveros VJ, Mizrahi I, Pilosof S. Multilayer networks of plasmid genetic similarity reveal potential pathways of gene transmission. ISME J. 2023;17(5):649–59. 36759552 10.1038/s41396-023-01373-5 PMC10119158

[7] de Nies L, Busi SB, Tsenkova M, Halder R, Letellier E, Wilmes P. Evolution of the murine gut resistome following broad-spectrum antibiotic treatment. Nat Commun. 2022;13(1):2296. 35484157 10.1038/s41467-022-29919-9 PMC9051133

[8] Busi SB, de Nies L, Habier J, Wampach L, Fritz JV, Heintz-Buschart A, May P, Halder R, de Beaufort C, Wilmes P. Persistence of birth mode-dependent effects on gut microbiome composition, immune system stimulation and antimicrobial resistance during the first year of life. ISME Commun. 2021;1(1):8. 36717704 10.1038/s43705-021-00003-5 PMC9723731

[9] Fishbein SRS, Mahmud B, Dantas G. Antibiotic perturbations to the gut microbiome. Nat Rev Microbiol. 2023;21(12):772–88. 37491458 10.1038/s41579-023-00933-y PMC12087466

[10] Scicchitano D, Leuzzi D, Babbi G, Palladino G, Turroni S, Laczny CC, Wilmes P, Correa F, Leekitcharoenphon P, Savojardo C. Dispersion of antimicrobial resistant bacteria in pig farms and in the surrounding environment. Anim Microbiome. 2024;6(1):17. 38555432 10.1186/s42523-024-00305-8 PMC10981832

[11] XXXX

[12] Kemp JO, Taylor JJ, Kelly LA, Larocque R, Heriazon A, Tiessen KH, Cooke SJ. Antibiotic resistance genes in the aquaculture sector: global reports and research gaps. Environ Rev. 2021;29(2):300–14.

[13] Sanches-Fernandes GM, Sá-Correia I, Costa R. Vibriosis outbreaks in aquaculture: addressing environmental and public health concerns and preventive therapies using gilthead seabream farming as a model system. Front Microbiol. 2022;13:904815. 35898915 10.3389/fmicb.2022.904815 PMC9309886

[14] Welch TJ, Evenhuis J, White DG, McDermott PF, Harbottle H, Miller RA, Griffin M, Wise D. IncA/C plasmid-mediated florfenicol resistance in the catfish pathogen *Edwardsiella ictaluri*. Antimicrob Agents Chemother. 2009;53(2):845–6. 19029319 10.1128/AAC.01312-08 PMC2630597

[15] Kalmar L, Gupta S, Kean IR, Ba X, Hadjirin N, Lay EM, de Vries SP, Bateman M, Bartlet H, Hernandez-Garcia J. HAM-ART: an optimised culture-free Hi-C metagenomics pipeline for tracking antimicrobial resistance genes in complex microbial communities. PLoS Genet. 2022;18(3):e1009776. 35286304 10.1371/journal.pgen.1009776 PMC8947609

[16] Wu R, Davison MR, Nelson WC, Smith ML, Lipton MS, Jansson JK, McClure RS, McDermott JE, Hofmockel KS. Hi-C metagenome sequencing reveals soil phage–host interactions. Nat Commun. 2023;14(1):7666. 37996432 10.1038/s41467-023-42967-z PMC10667309

[17] Yaffe E, Relman DA. Tracking microbial evolution in the human gut using Hi-C reveals extensive horizontal gene transfer, persistence and adaptation. Nat Microbiol. 2020;5(2):343–53. 31873203 10.1038/s41564-019-0625-0 PMC6992475

[18] Calderón-Franco D, Van Loosdrecht MC, Abeel T, Weissbrodt DG. Catch me if you can: capturing microbial community transformation by extracellular DNA using Hi-C sequencing. Antonie Van Leeuwenhoek. 2023;116(7):667–85. 37156983 10.1007/s10482-023-01834-z PMC10257627

[19] Hilpert C, Bricheux G, Debroas D. Reconstruction of plasmids by shotgun sequencing from environmental DNA: which bioinformatic workflow? Brief Bioinform. 2021;22(3):bbaa059. 32427283 10.1093/bib/bbaa059

[20] Schuele L, Fleres G, Strutzberg-Minder K, Schütze S, Löbert S, Lambrecht C, Harlizius J, Peter S, Rossen JW, Couto N. Detection of a small IncX4 plasmid carrying the mcr-1.1 gene in a pig oral fluid sample by shotgun metagenomic sequencing. J Glob Antimicrob Resist. 2021;24:205–6. 33482366 10.1016/j.jgar.2020.12.020

[21] Alvarez-Garcia V, Bartos C, Keraite I, Trivedi U, Brennan PM, Kersaudy-Kerhoas M, Gharbi K, Oikonomidou O, Leslie NR. A simple and robust real-time qPCR method for the detection of PIK3CA mutations. Sci Rep. 2018;8(1):4290. 29523855 10.1038/s41598-018-22473-9 PMC5844869

[22] Kazlauskaite R, Cheaib B, Heys C, Ijaz UZ, Connelly S, Sloan W, Russel J, Rubio L, Sweetman J, Kitts A. SalmoSim: the development of a three-compartment in vitro simulator of the Atlantic salmon GI tract and associated microbial communities. Microbiome. 2021;9:1–20. 34465363 10.1186/s40168-021-01134-6 PMC8408954

[23] Kazlauskaite R, Cheaib B, Humble J, Heys C, Ijaz UZ, Connelly S, Sloan WT, Russell J, Martinez-Rubio L, Sweetman J. Deploying an in vitro gut model to assay the impact of the mannan-oligosaccharide prebiotic Bio-Mos on the Atlantic salmon (*Salmo salar*) gut microbiome. Microbiol Spectr. 2022;10(3):e01953-1921. 35532227 10.1128/spectrum.01953-21 PMC9241627

[24] Ferrières L, Hémery G, Nham T, Guérout AM, Mazel D, Beloin C, Ghigo JM. Silent mischief: bacteriophage Mu insertions contaminate products of *Escherichia coli* random mutagenesis performed using suicidal transposon delivery plasmids mobilized by broad-host-range RP4 conjugative machinery. J Bacteriol. 2010;192(24):6418–27. 20935093 10.1128/JB.00621-10 PMC3008518

[25] Minogue T, Daligault H, Davenport KW, Bishop-Lilly K, Broomall S, Bruce DC, Chain P, Chertkov O, Coyne S, Freitas T. Complete genome assembly of *Escherichia coli* ATCC 25922, a serotype O6 reference strain. Genome Announc. 2014. 10.1128/genomea.00969-00914. 25291776 10.1128/genomeA.00969-14 PMC4175212

[26] Marlène M, Lee B, Miller S, Zeferino R, Lu P, Yan J, Amber U, Skandalis N, Brad S, Luna B. Horizontal gene transfer of antibiotic resistance from *Acinetobacter baylyi* to *Escherichia coli* on lettuce and subsequent antibiotic resistance transmission to the gut microbiome. mSphere. 2020;5:(3). 10.1128/mSphere.00329-20 PMC7253597 32461272

[27] Lieberman-Aiden E, Van Berkum NL, Williams L, Imakaev M, Ragoczy T, Telling A, Amit I, Lajoie BR, Sabo PJ, Dorschner MO. Comprehensive mapping of long-range interactions reveals folding principles of the human genome. Science. 2009;326(5950):289–93. 19815776 10.1126/science.1181369 PMC2858594

[28] ZymoBIOMICS DNA Miniprep Kit. https://www.zymoresearch.com/products/zymobiomics-dna-miniprep-kit

[29] Chen S, Zhou Y, Chen Y, Gu J. fastp: an ultra-fast all-in-one FASTQ preprocessor. Bioinformatics. 2018;34(17):i884–90. 30423086 10.1093/bioinformatics/bty560 PMC6129281

[30] Li D, Liu C-M, Luo R, Sadakane K, Lam T-W. MEGAHIT: an ultra-fast single-node solution for large and complex metagenomics assembly via succinct de Bruijn graph. Bioinformatics. 2015;31(10):1674–6. 25609793 10.1093/bioinformatics/btv033

[31] Li D, Luo R, Liu C-M, Leung C-M, Ting H-F, Sadakane K, Yamashita H, Lam T-W. MEGAHIT v1. 0: a fast and scalable metagenome assembler driven by advanced methodologies and community practices. Methods. 2016;102:3–11. 27012178 10.1016/j.ymeth.2016.02.020

[32] Aligning and QCing Phase Genomics Hi-C Data [https://phasegenomics.github.io/2019/09/19/hic-alignment-and-qc.html]

[33] Li H, Durbin R. Fast and accurate long-read alignment with Burrows-Wheeler transform. Bioinformatics. 2010;26(5):589–95. 20080505 10.1093/bioinformatics/btp698 PMC2828108

[34] Faust GG, Hall IM. SAMBLASTER: fast duplicate marking and structural variant read extraction. Bioinformatics. 2014;30(17):2503–5. 24812344 10.1093/bioinformatics/btu314 PMC4147885

[35] Li H, Handsaker B, Wysoker A, Fennell T, Ruan J, Homer N, Marth G, Abecasis G, Durbin R, Subgroup GPDP. The sequence alignment/map format and SAMtools. Bioinformatics. 2009;25(16):2078–9. 19505943 10.1093/bioinformatics/btp352 PMC2723002

[36] Press MO, Wiser AH, Kronenberg ZN, Langford KW, Shakya M, Lo C-C, Mueller KA, Sullivan ST, Chain PS, Liachko I: Hi-C deconvolution of a human gut microbiome yields high-quality draft genomes and reveals plasmid-genome interactions. *biorxiv* 2017:198713.

[37] Stewart RD, Auffret MD, Warr A, Wiser AH, Press MO, Langford KW, Liachko I, Snelling TJ, Dewhurst RJ, Walker AW. Assembly of 913 microbial genomes from metagenomic sequencing of the cow rumen. Nat Commun. 2018;9(1):870. 29491419 10.1038/s41467-018-03317-6 PMC5830445

[38] Parks DH, Imelfort M, Skennerton CT, Hugenholtz P, Tyson GW. CheckM: assessing the quality of microbial genomes recovered from isolates, single cells, and metagenomes. Genome Res. 2015;25(7):1043–55. 25977477 10.1101/gr.186072.114 PMC4484387

[39] Ondov BD, Treangen TJ, Melsted P, Mallonee AB, Bergman NH, Koren S, Phillippy AM. Mash: fast genome and metagenome distance estimation using MinHash. Genome Biol. 2016;17:1–14. 27323842 10.1186/s13059-016-0997-x PMC4915045

[40] Bankevich A, Nurk S, Antipov D, Gurevich AA, Dvorkin M, Kulikov AS, Lesin VM, Nikolenko SI, Pham S, Prjibelski AD. SPAdes: a new genome assembly algorithm and its applications to single-cell sequencing. J Comput Biol. 2012;19(5):455–77. 22506599 10.1089/cmb.2012.0021 PMC3342519

[41] Alcock BP, Huynh W, Chalil R, Smith KW, Raphenya AR, Wlodarski MA, Edalatmand A, Petkau A, Syed SA, Tsang KK. CARD 2023: expanded curation, support for machine learning, and resistome prediction at the comprehensive antibiotic resistance database. Nucleic Acids Res. 2023;51(D1):D690–9. 36263822 10.1093/nar/gkac920 PMC9825576

[42] Sumithra T, Sharma KS, Gangadharan S, Suresh G, Prasad V, Amala P, Sayooj P, Gop AP, Anil M, Patil PK. Dysbiosis and restoration dynamics of the gut microbiome following therapeutic exposure to florfenicol in snubnose pompano (*Trachinotus blochii*) to aid in sustainable aquaculture production strategies. Front Microbiol. 2022;13:881275. 35707172 10.3389/fmicb.2022.881275 PMC9189426

[43] Shiroma LS, Soares MP, Cardoso IL, Ishikawa MM, Jonsson CM, Queiroz SCN. Evaluation of health and environmental risks for juvenile tilapia (*Oreochromis niloticus*) exposed to florfenicol. Heliyon. 2020;6:(12). 10.1016/j.heliyon.2020.e05716 PMC7750370 33364491

[44] Zhang Z, Yang Q, Xu W, Tang R, Li L, Li D. Short-term feeding of dietary florfenicol influences gut microbiome and growth performance of fast-growing *Silurus meridionalis*. Aquaculture. 2023;574:739645.

[45] Florfenicol (Aquaflor®): U.S. Fish & Wildlife Service. https://www.fws.gov/inad/florfenicol-aquaflorr-10-697

[46] Merck-animal-health-USA. https://www.merck-animal-health-usa.com/offload-downloads/univaxbd-product-bulletin

[47] Wilpiszeski RL, Gionfriddo CM, Wymore AM, Moon J-W, Lowe KA, Podar M, Rafie S, Fields MW, Hazen TC, Ge X. In-field bioreactors demonstrate dynamic shifts in microbial communities in response to geochemical perturbations. PLoS ONE. 2020;15(9):e0232437. 32986713 10.1371/journal.pone.0232437 PMC7521895

[48] Philippot L, Griffiths BS, Langenheder S. Microbial community resilience across ecosystems and multiple disturbances. Microbiol Mol Biol Rev. 2021. 10.1128/mmbr.00026-00020. 33789927 10.1128/MMBR.00026-20 PMC8139522

[49] Ekhlas D, Cobo Díaz JF, Cabrera-Rubio R, Alexa E, Ortiz Sanjuán JM, Garcia Manzanilla E, Crispie F, Cotter PD, Leonard FC, Argüello H. Metagenomic comparison of the faecal and environmental resistome on Irish commercial pig farms with and without zinc oxide and antimicrobial usage. Anim Microbiome. 2023;5(1):62. 38082336 10.1186/s42523-023-00283-3 PMC10712031

[50] Trieu-Cuot P, Carlier C, Martin P, Courvalin P. Plasmid transfer by conjugation from *Escherichia coli* to gram-positive bacteria. FEMS Microbiol Lett. 1987;48(1–2):289–94.

[51] Schäfer A, Kalinowski J, Simon R, Seep-Feldhaus A, Pühler A. High-frequency conjugal plasmid transfer from gram-negative *Escherichia coli* to various gram-positive coryneform bacteria. J Bacteriol. 1990;172(3):1663–6. 2106514 10.1128/jb.172.3.1663-1666.1990 PMC208647

[52] Pinilla-Redondo R, Olesen AK, Russel J, de Vries LE, Christensen LD, Musovic S, Nesme J, Sørensen SJ. Broad dissemination of plasmids across groundwater-fed rapid sand filter microbiomes. MBio. 2021;12(6):e03068-e13021. 34844427 10.1128/mBio.03068-21 PMC8630534

[53] Klümper U, Riber L, Dechesne A, Sannazzarro A, Hansen LH, Sørensen SJ, Smets BF. Broad host range plasmids can invade an unexpectedly diverse fraction of a soil bacterial community. ISME J. 2015;9(4):934–45. 25333461 10.1038/ismej.2014.191 PMC4817699

[54] Yang QE, Ma X, Zeng L, Wang Q, Li M, Teng L, He M, Liu C, Zhao M, Wang M. Interphylum dissemination of NDM-5-positive plasmids in hospital wastewater from Fuzhou, China: a single-centre, culture-independent, plasmid transmission study. Lancet Microbe. 2024;5(1):e13–23. 38006896 10.1016/S2666-5247(23)00227-6

[55] Gueimonde M, Sánchez BG, de los Reyes-Gavilán C, Margolles A. Antibiotic resistance in probiotic bacteria. Front Microbiol. 2013;4:202. 23882264 10.3389/fmicb.2013.00202 PMC3714544

[56] He D-D, Cui M-M, Zhang T-L, Hu G-Z, Liu J-H, Pan Y-S. Characterization of blaCMY-2-carrying IncC and rmtB-carrying IncI1/ST136 plasmids in an avian *Escherichia coli* ST224 strain. Plasmid. 2021;114:102555. 33472047 10.1016/j.plasmid.2021.102555

[57] Darphorn TS, Bel K, Koenders-van Sint Anneland BB, Brul S, Ter-Kuile BH. Antibiotic resistance plasmid composition and architecture in *Escherichia coli* isolates from meat. Sci Rep. 2021;11(1):2136. 33483623 10.1038/s41598-021-81683-w PMC7822866

[58] Ma T, Zaheer R, McAllister TA, Guo W, Li F, Tu Y, Diao Q, Guan LL. Expressions of resistome is linked to the key functions and stability of active rumen microbiome. Anim Microbiome. 2022;4(1):38. 35659381 10.1186/s42523-022-00189-6 PMC9167530

[59] Lund P, Tramonti A, De Biase D. Coping with low pH: molecular strategies in neutralophilic bacteria. FEMS Microbiol Rev. 2014;38(6):1091–125. 24898062 10.1111/1574-6976.12076

[60] Guardiola FA, Esteban MÁ, Angulo C. *Yarrowia lipolytica*, health benefits for animals. Appl Microbiol Biotechnol. 2021;105(20):1–16. 10.1007/s00253-021-11584-5 34536101

[61] Singh A, Vidakovic A, Singh A, Dicksved J, Schnurer A, Lundh T. *Yarrowia lipolytica* yeast as a dietary supplement for rainbow trout (*Oncorhynchus mykiss*): effects on gut microbiota, health and immunity. Aquaculture. 2024;590:741065.

[62] Timoumi A, Cléret M, Bideaux C, Guillouet SE, Allouche Y, Molina-Jouve C, Fillaudeau L, Gorret N. Dynamic behavior of *Yarrowia lipolytica* in response to pH perturbations: dependence of the stress response on the culture mode. Appl Microbiol Biotechnol. 2017;101:351–66. 27730339 10.1007/s00253-016-7856-2

[63] Celińska E. “Fight-flight-or-freeze”—how *Yarrowia lipolytica* responds to stress at molecular level? Appl Microbiol Biotechnol. 2022;106(9):3369–95. 35488934 10.1007/s00253-022-11934-x PMC9151528

[64] Risely A, Newbury A, Stalder T, Simmons BI, Top EM, Buckling A, Sanders D. Host-plasmid network structure in wastewater is linked to antimicrobial resistance genes. Nat Commun. 2024;15(1):555. 38228585 10.1038/s41467-024-44827-w PMC10791616

[65] Du Y, Laperriere SM, Fuhrman J, Sun F. Normalizing metagenomic Hi-C data and detecting spurious contacts using zero-inflated negative binomial regression. J Comput Biol. 2022;29(2):106–20. 35020412 10.1089/cmb.2021.0439 PMC8892984

[66] Rojas CA, Ganz HH, Gardy J, Eisen JA. Recovery of high-quality genomes from the fecal microbiome of the domestic cat (*Felis catus*) using Hi-C proximity ligation. 2022. 10.21203/rs.3.rs-2274246/v1. 10.1128/MRA.00601-23 PMC10586161 37695121

[67] Bush K. Past and present perspectives on β-lactamases. Antimicrob Agents Chemother. 2018;62:(10). 10.1128/AAC.01076-18 PMC6153792 30061284

[68] Zheng D, Bergen PJ, Landersdorfer CB, Hirsch EB. Differences in fosfomycin resistance mechanisms between *Pseudomonas aeruginosa* and Enterobacterales. Antimicrob Agents Chemother. 2022;66(2):e01446-e11421. 34807759 10.1128/AAC.01446-21 PMC8846481

[69] Wang Y, Lu J, Engelstädter J, Zhang S, Ding P, Mao L, Yuan Z, Bond PL, Guo J. Correction: non-antibiotic pharmaceuticals enhance the transmission of exogenous antibiotic resistance genes through bacterial transformation. ISME J. 2022;16(2):612–612. 34341509 10.1038/s41396-021-01074-x PMC8776780

[70] Schneider CL. Bacteriophage-mediated horizontal gene transfer: transduction. Bacteriophages Biol Technol Ther. 2021;151–92. 10.1007/978-3-319-41986-2_4.

[71] Wang Y, Batra A, Schulenburg H, Dagan T. Gene sharing among plasmids and chromosomes reveals barriers for antibiotic resistance gene transfer. Philos Trans R Soc B. 1842;2022(377):20200467. 10.1098/rstb.2020.0467 PMC8628082 34839702

[72] Cronin M: The usage of antibiotics and their alternatives in Scottish salmon farms. 2023.

[73] Bondad-Reantaso MG, MacKinnon B, Karunasagar I, Fridman S, Alday-Sanz V, Brun E, Le Groumellec M, Li A, Surachetpong W, Karunasagar I. Review of alternatives to antibiotic use in aquaculture. Rev Aquac. 2023;15(4):1421–51.

[74] Lulijwa R, Rupia EJ, Alfaro AC. Antibiotic use in aquaculture, policies and regulation, health and environmental risks: a review of the top 15 major producers. Rev Aquac. 2020;12(2):640–63.

