# Supplementary material for: Understanding the transfer and persistence of antimicrobial resistance in aquaculture using a model teleost gut system
Source: Anim Microbiome. 2025 Feb 22;7:18. doi: 10.1186/s42523-025-00377-0 (PMC11846170; doi:10.1186/s42523-025-00377-0)
Supplement: Supplementary file 1 — Additional file 1. [file 42523_2025_377_MOESM1_ESM.docx]

**Supplementary material**

The table includes the relative abundance of each genus, normalized as percentages of the total read counts in each bioreactor. Additionally, we have highlighted in red the genera and corresponding bioreactors where plasmid transfer occurred, making the connection between microbial interactions and AMR transfer more explicit. Genera with non-zero relative abundance values are included to ensure clarity and relevance.

| Table S1. Comprehensive Comparison of Genera Across Bioreactors with Emphasis on Plasmid Transfer and AMR Interactions | | | |
| --- | --- | --- | --- |
| Genus | Relative Abundance in A (%) | Relative Abundance in B (%) | Relative Abundance in C (%) |
| Bacillus | 41.39 | 2.4 | 12.01 |
| Cytobacillus | 6.25 | 16.88 | 13.7 |
| Photobacterium | 7.43 | 8.29 | 11.57 |
| Metabacillus | 0 | 24.99 | 0 |
| Psychrobacter | 0.96 | 3.55 | 22.47 |
| Stenotrophomonas | 1.55 | 9.65 | 5.61 |
| Yarrowia | 21.63 | 0.39 | 0.63 |
| Escherichia | 12.54 | 2.5 | 2.84 |
| Candida | 0 | 3.31 | 10.06 |
| Aliivibrio | 1.84 | 2.45 | 6.47 |
| Brachybacterium | 0 | 8.14 | 0 |
| Pyricularia | 0.13 | 0 | 0 |
| Microbacterium | 0 | 6.46 | 0 |
| Fusarium | 0 | 0 | 0.11 |
| Enterococcus | 0.08 | 0.16 | 6.11 |
| Vibrio | 0.64 | 0.59 | 2.13 |
| Brevibacterium | 0 | 3.16 | 0 |
| Aspergillus | 0 | 0 | 0.04 |
| Micrococcus | 3.06 | 0.33 | 0 |
| Lodderomyces | 0 | 0.12 | 0.76 |
| Streptomyces | 0.05 | 0.59 | 0.08 |
| Arthrobacter | 0 | 1.63 | 0 |
| Marinilactibacillus | 0.8 | 0.74 | 0.23 |
| Lysinibacillus | 0 | 0.52 | 0.72 |
| Scheffersomyces | 0 | 0 | 1.1 |
| Clostridium | 0 | 0.03 | 0 |
| Dermacoccus | 1.51 | 0 | 0 |
| Staphylococcus | 0.05 | 0.06 | 0 |
| Pseudomonas | 0.04 | 0.07 | 0.1 |
| Acinetobacter | 0 | 0 | 0.35 |
| Clavispora | 0 | 0 | 0.61 |
| Saccharomyces | 0 | 0.04 | 0.29 |
| Glutamicibacter | 0 | 0.5 | 0 |
| Eremothecium | 0 | 0 | 0.31 |
| Kluyveromyces | 0 | 0 | 0.2 |
| Paenibacillus | 0 | 0.09 | 0 |
| Pseudoalteromonas | 0 | 0.02 | 0.08 |
| Debaryomyces | 0 | 0.05 | 0.47 |
| Streptococcus | 0.04 | 0.08 | 0 |
| Shewanella | 0 | 0.04 | 0.11 |
| Corynebacterium | 0 | 0.12 | 0 |
| Kocuria | 0 | 0.22 | 0 |
| Tetrapisispora | 0 | 0 | 0.1 |
| Priestia | 0 | 0.12 | 0 |
| Pseudarthrobacter | 0 | 0.2 | 0 |
| Naumovozyma | 0 | 0 | 0.05 |
| Mycolicibacterium | 0 | 0.09 | 0 |
| Kazachstania | 0 | 0 | 0.13 |
| Neobacillus | 0 | 0.1 | 0 |
| Torulaspora | 0 | 0 | 0.14 |
| Curtobacterium | 0 | 0.11 | 0 |
| Burkholderia | 0 | 0.02 | 0 |
| Rhodococcus | 0 | 0.09 | 0 |
| Amycolatopsis | 0 | 0.03 | 0 |
| Acidovorax | 0 | 0.03 | 0 |
| Lactobacillus | 0 | 0.04 | 0 |
| Peribacillus | 0 | 0.08 | 0 |
| Sugiyamaella | 0 | 0 | 0.03 |
| Brettanomyces | 0 | 0 | 0.06 |
| Nocardioides | 0 | 0.09 | 0 |
| Carnobacterium | 0.04 | 0.06 | 0.02 |
| Moraxella | 0 | 0 | 0.1 |
| Alkalibacterium | 0.02 | 0.04 | 0.03 |
| Mycobacterium | 0 | 0.03 | 0 |
| Listeria | 0 | 0 | 0.07 |
| Xanthomonas | 0 | 0.02 | 0 |
| Moritella | 0 | 0.02 | 0.03 |
| Vagococcus | 0 | 0.04 | 0 |
| Nakaseomyces | 0 | 0 | 0.04 |
| Actinomyces | 0 | 0.05 | 0 |
| Nocardia | 0 | 0.04 | 0 |
| Gordonia | 0 | 0.08 | 0 |
| Micromonospora | 0 | 0.03 | 0 |
| Paenarthrobacter | 0 | 0.06 | 0 |
| Ogataea | 0 | 0 | 0.03 |
| Pseudonocardia | 0 | 0.03 | 0 |
| Rothia | 0 | 0.05 | 0 |
| Bifidobacterium | 0 | 0.02 | 0 |
| Komagataella | 0 | 0 | 0.06 |
| Citricoccus | 0 | 0.04 | 0 |
| Desemzia | 0 | 0.04 | 0 |
| Sinomonas | 0 | 0.04 | 0 |
| Helcobacillus | 0 | 0.03 | 0 |
| Neomicrococcus | 0 | 0.03 | 0 |
| Cryobacterium | 0 | 0.03 | 0 |
| Leucobacter | 0 | 0.03 | 0 |
| Lachancea | 0 | 0 | 0.03 |
| Cellulomonas | 0 | 0.02 | 0 |
| Kytococcus | 0 | 0.02 | 0 |
| Agromyces | 0 | 0.02 | 0 |
| Rathayibacter | 0 | 0.02 | 0 |
